# Hyperbaric oxygen preconditioning attenuates hyperglycemia enhanced hemorrhagic transformation after transient MCAO in rats

**DOI:** 10.1186/2045-9912-2-9

**Published:** 2012-04-11

**Authors:** Yoshiteru Soejima, Robert P Ostrowski, Anatol Manaenko, Mutsumi Fujii, Jiping Tang, John H Zhang

**Affiliations:** 1 Department of Physiology and Pharmacology, Loma Linda University School of Medicine, Loma Linda, CA, USA

**Keywords:** Hemorrhagic transformation, MCAO, Hyperbaric oxygen preconditioning, Nuclear factor erythroid 2-related factor 2 (Nrf2), Heme oxygenase-1 (HO-1)

## Abstract

**Background:**

Hemorrhagic transformation (HT) can be a devastating complication of ischemic stroke. Hyperbaric oxygen preconditioning (HBO-PC) has been shown to improve blood-brain barrier (BBB) permeability in stroke models. The purpose of this study is to examine whether HBO-PC attenuates HT after focal cerebral ischemia, and to investigate whether the mechanism of HBO-PC against HT includes up-regulation of antioxidants in hyperglycemic rats.

**Methods:**

Male Sprague-Dawley rats (280-320 g) were divided into the following groups: sham, middle cerebral artery occlusion (MCAO) for 2 h, and MCAO treated with HBO-PC. HBO-PC was conducted giving 100% oxygen at 2.5 atm absolute (ATA), for 1 h at every 24 h interval for 5 days. At 24 h after the last session of HBO-PC, rats received an injection of 50% glucose (6 ml/kg intraperitoneally) and were subjected to MCAO 15 min later. At 24 h after MCAO, neurological behavior tests, infarct volume, blood-brain barrier permeability, and hemoglobin content were measured to evaluate the effect of HBO-PC. Western blot analysis of nuclear factor erythroid 2-related factor 2 (Nrf2) and heme oxygenase-1 (HO-1) was evaluated at multiple time-points before and after MCAO.

**Results:**

HBO-PC improved neurological behavior test, and reduced infarction volume, HT and Evans blue extravasation in the ipsilateral hemisphere at 24 h after MCAO. Western blot analysis failed to demonstrate up-regulation of Nrf2 in HBO-PC group before and after MCAO. Paradoxically, HBO-PC decreased HO-1 expression at 24 h after MCAO, as compared with htMCAO group.

**Conclusions:**

HBO-PC improved neurological deficits, infarction volume, BBB disruption, and HT after focal cerebral ischemia. However, its mechanism against focal cerebral ischemia and HT may not include activation of Nrf2 and subsequent HO-1 expression.

## Background

Hemorrhagic transformation (HT) of ischemic stroke contributes largely to the early mortality and poor functional recovery of affected patients. Experimental studies have shown that preischemic hyperglycemia increases the risk of bleeding into cerebral infarction area, which aggravates brain damage after reperfusion [1]. Hyperglycemia-enhanced HT may be linked to increased activity of inflammation and oxidative stress, which cause blood-brain barrier (BBB) disruption and neuronal cell death [2]. Oxidative stress leading to ischemic cell death involves the formation of reactive oxygen species/reactive nitrogen species (ROS/RNS), which induce BBB disruption through degradation of junctional proteins composing tight junction [3,4].

Nuclear factor erythroid 2-related factor 2 (Nrf2), a cap 'n' Collar transcription factor, regulates a lot of antioxidant/detoxification genes acting in synergy to remove ROS/RNS through sequential enzymatic reactions [5,6]. Under non-stressed conditions, Nrf2 interacts with Kelch-like ECH-associated protein 1 (Keap l) to form the Keap 1-Nrf2 complex that limits Nrf2-mediated gene expression. Upon activation, the Keap 1-Nrf2 complex is dissociated, Nrf2 translocates into the nuclei to bind antioxidant response element (ARE) and activates ARE-dependent transcription of important antioxidant and detoxification genes (Phase II genes) [5]. Phase II genes, including heme oxygenase-1 (HO-1), glutathione S-transferases (GSTs) and NAD(P)H quinone oxidoreductase, work in synergy to constitute a pleiotropic cellular defense that scavenges ROS/RNS, detoxifies electrophiles and xenobiotics, maintain intracellular reducing potential [7,8]. HO-1 is a ubiquitous and redox-sensitive inducible stress protein that degrades heme to carbon monooxide, iron and biliverdin [9]. All products of heme oxygenases exert strong antioxidant, anti-inflammatory and antiapoptotic effects.

Hyperbaric oxygen preconditioning (HBO-PC) induces tolerance against brain ischemia reperfusion injury by up-regulation of antioxidant enzymes [10], as well as ischemia tolerance in organs including the spinal cord [11], myocardium [12], and liver [13]. Recent investigation indicated that HBO-PC could induce cytoprotective effect on human microvascular endothelial cell via upregulation of Nrf2 and HO-1 [14]. The purpose of this study is to examine whether HBO-PC attenuates HT after focal cerebral ischemia, and to investigate whether the mechanism of HBO-PC against HT includes Nrf2/HO-1 up-regulation in hyperglycemic rats.

## Methods

### Animal preparation and MCAO

All experiments were approved by the Institutional Animal Care and Use Committee of Loma Linda University; 96 male Sprague-Dawley rats were purchased from Harlan Laboratories (Indianapolis, IN) and randomly divided into the following groups: middle cerebral artery occlusion (MCAO) with hemorrhagic transformation (htMCAO; n = 40), HBO preconditioned htMCAO (HBO-PC; n = 40), and sham-operated group (sham; n = 16) for analysis of Evans blue extravasation and Western blot. All rats received 50% dextrose (6 ml/kg) intraperitoneally 30 min before MCAO to induce acute hyperglycemia. Anesthesia was induced with ketamine and xylazine (80 mg/kg and 10 mg/kg respectively, intraperitoneally), followed by atropine at a dose of 0.1 mg/kg subcutaneously. During surgery and postoperative period, rectal temperature was maintained at 37.0°C by using a feedback-controlled heating pad.

MCAO was performed as reported previously [15]. Briefly, the right external carotid artery was isolated and coagulated. A 4-0 nylon suture with a round tip was inserted into the internal carotid artery through the external carotid artery stump and advanced to occlude the origine of MCA. The suture was removed at 2 h after occlusion. Blood was obtained from the tail vein for analysis of glucose level at multiple time-points.

### HBO-PC regimen

Rats were pressurized in a research hyperbaric chamber (1300B; Sechrist) at 2.5 atm absolutes with 100% oxygen (flow of 22 L/min). Compression and decompression were maintained at a rate of 5 psi/min. A 1-h HBO session was administered daily for 5 consecutive days; the last session was performed 24 h before MCAO.

### 2,3,5-triphenyltetrazolium chloride staining and evaluation of infarction volume

As previously reported [15], 2,3,5-triphenyltetrazolium chloride monohydrate (TTC) staining was performed to determine the infarct volume at 24 h after MCAO. The possible interference of brain edema with infarct volume was corrected by standard methods (whole contralateral hemisphere volume - nonischemic ipsilateral hemisphere volume) and the infarct volume was expressed as a ratio of infarct volume to the whole contralateral hemisphere [16].

### Spectrophotometric assay of hemoglobin

Hemorrhagic transformation was quantified with spectrophotometric assay of brain hemoglobin content [17]. At 24 h after MCAO (22 h after reperfusion) or sham-operation, the animals were perfused transcardially with 0.1 mol/l phosphate-buffered saline under deep anesthesia until the outflow fluid from the right atrium was colorless. The brain was rapidly removed and dissected into the left hemisphere and the right hemisphere.

Cerebral hemorrhage was quantified using a previously described spectrophotometric assay with some modifications [17]. A standard curve was obtained using a "virtual" model of hemorrhage. Incremental volumes of homologous blood (0, 2, 4, 8, 16, 32 μl) were added to the perfused brain tissue. The hemispheric brain tissue was then homogenized in distilled water followed by 30-min centrifugation (13,000 g). Drabkin reagent (1.6 ml; Sigma) was added to 0.4 ml supernatant aliquots and optical density was measured at 540 nm via spectrophotometer (Spectronix 3000; Milton-Roy). Hemoglobin measurements were performed and compared with the standard curve to obtain data in terms of hemorrhage volume. The total hemispheric hemoglobin content was expressed as μl of blood per hemisphere.

### Measurement of evans blue dye extravasation

The integrity of the BBB was investigated by measuring the extravasation of Evans blue in sham, htMCAO, and HBO-PC group (n = 5 each). Evans blue dye (2% in saline, 5 ml/kg) was injected intravenously at 23 h after operation. One hour after Evans blue injection, the chest wall was opened under deep anesthesia and animals were perfused with 0.1 mol/l phosphate-buffered saline through the left ventricle to remove the intravascular localized dye until colorless perfusion fluid was obtained from the right atrium. After decapitation, brains were removed, weighed, and homogenized in 1.0 ml of 0.1 mol/l phosphate-buffered saline, and centrifuged at 15,000 rpm for 30 min. Then 0.6 ml of the resultant supernatant was added to an equal volume of trichloroacetic acid. After overnight incubation at 4°C and centrifugation at 15,000 rpm for 30 min, the supernatant was measured at 615 nm for absorbance using a spectrophotometer (pectronix 3000; Milton-Roy). The tissue content of Evans blue was quantified from a linear standard curve and was expressed as micrograms per gram of brain tissue [18].

### Neurological scores

At 24 h after MCAO, a neurological examination was performed by a blinded investigator as previously described with modifications [19]. The scores given to each rat at the completion of the evaluation was the summation of all 7 individual test scores [20].

### Western blot analysis

The animals were euthanized under general anesthesia at 24 h after operation (n = 6, each group), 2 and 24 h after the last HBO session (n = 5, each). Brains were collected and protein extracts from nuclear and cytosolic fraction were obtained using a nuclear extraction kit and following the manufacturer protocol (Millipore). Equal amounts of total protein (20 μg) were separated in 10% SDS-PAGE and blotted onto nitrocellulose membranes. The probing antibodies included polyclonal rabbit anti-Nrf2 antibody (1:1000; Abcam Inc), rabbit anti-heme oxygenase 1 (HO-1) polyclonal antibody (1:1000; Enzo Life Sciences), goat anti-actin (1:2000; Santa Cruz), and mouse anti-histone H1 monoclonal antibody (1:500; Millipore Inc). Bands were detected by ECL plus Western blotting detection kit (GE Healthcare) and recorded on X-ray film (Kodak). Bands were quantified by optical density method using Image J software, and densities were expressed relative to histone H1 (nuclear fraction), actin (cytosolic fraction) or sham.

### Statistical analyses

Data were expressed as the mean ± SEM. Statistical differences among groups were analyzed by using one-way analysis of variance followed by the Turkey method. Comparisons between the two groups for the infarction volume, hemorrhagic volume and the neurological scores were assessed by the unpaired *t*-test, and for mortality rate by chi-square test. *P *< 0.05 was considered statistically significant.

## Results

### Blood glucose level

**Figure 1:**
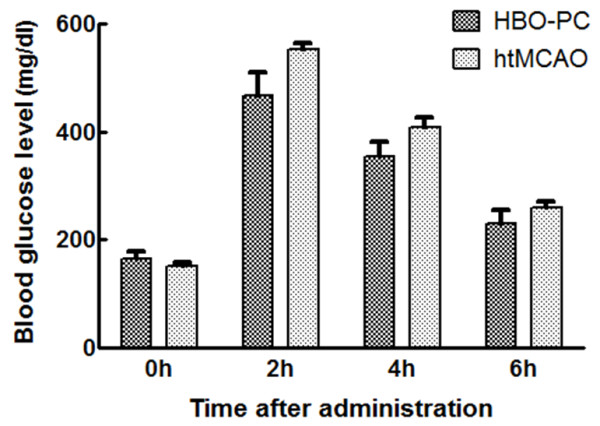
**Hyperbarc preconditioning had no effect on blood glcose levels at 4 time-points**. Blood was obtained from the tail vein for analysis of blood glcose before, 2 h, 4 h, and 6 h after administration of dextrose. There were not significant differences in the time course of blood glucose levels between HBO-PC group and htMCAO group.

The glucose levels at 2 h after injection in both groups were significantly higher than the baseline and high glucose level was lasted by 6 h after injection. HBO-PC had no effects on the blood glucose level (Figure 1).

### HBO-PC attenuated the outcomes of hyperglycemia induced HT at 24 h after MCAO

**Figure 2:**
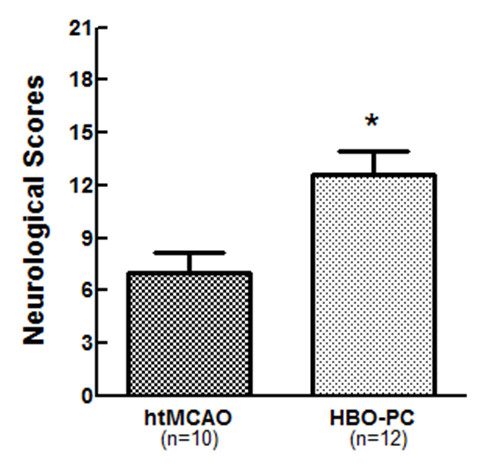
**HBO preconditioning significantly improved neurological outcome**. Modified Garcia score was used. The minimum neurological score (most severe) was 3, and the maximum was 21. HBO preconditioning significantly improved the postischemic neurological deficit at 24 h after MCAO.

**Figure 3:**
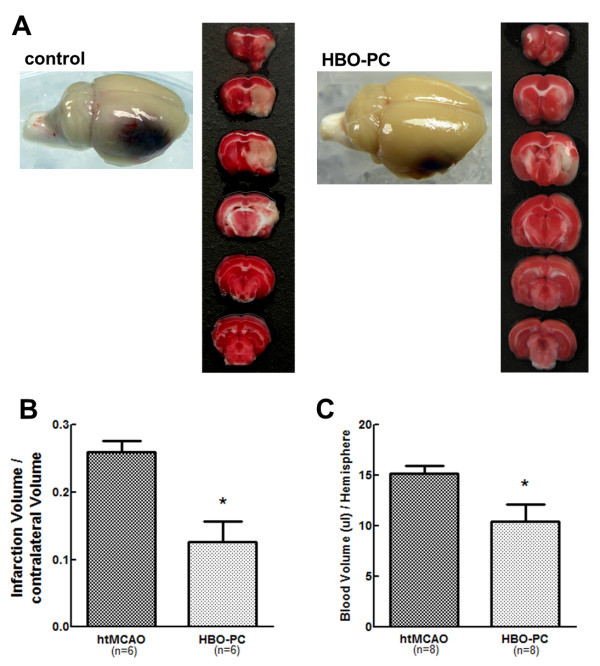
**HBO preconditioning significantly reduced infarction volume and hemorrhage volume**. (A) Representative whole brains and brain slices with 2,3,5-triphenyltetrazolium chloride staining of each groups were demonstrated. HBO-PC reduced infarction volume and attenuated hemorrhagic transformation. (B) Bar graphs showing average infarct size calculated in six slices. HBO-PC significantly reduced infarct volume at 24 h after reperfusion compared to htMCAO group. (C) Hemorrhage volume was significantly reduced with HBO-PC. *P > 0.05 vs. htMCAO.

To examine whether HBO-PC exerts a beneficial effect on HT after focal ischemia, neurological scores, infarction volume and hemorrhagic volume were evaluated in htMCAO and HBO-PC group. HBO preconditioned animals showed statistically better neurological function as compared with those of htMCAO group (7.00 ± 1.55 versus 12.58 ± 1.36, *P *< 0.01; Figure 2). HBO-PC significantly reduced the infarction volume on TTC staining at 24 h after MCAO (0.260 ± 0.017 versus 0.126 ± 0.031, *P *< 0.01; Figure 3A, B). Spectrophotometric measurement of brain hemoglobin showed that hemoglobin contents of HBO preconditioned animals were significantly lower than those of htMCAO group (10.37 ± 1.69 versus 15.17 ± 0.76 μL/hemisphere, *P *< 0.05; Figure 3C).

**Figure 4:**
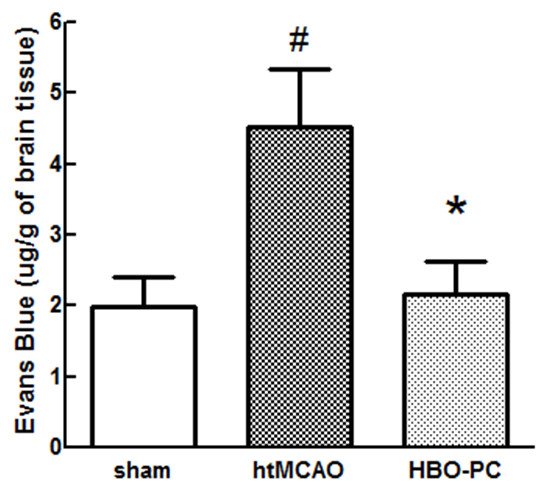
**HBO preconditioning significantly reduced Evans blue extravasation**. Animals were injected with Evans blue 23 h after MCAO. At 1 h after injection, animals were sacrificed after cardiac perfusion and quantitative evaluation analysis was evaluated. Extravasated Evans blue dye was significantly reduced by HBO-PC. *P > 0.05 vs. htMCAO, ^#^P > 0.05 vs. sham.

Evans blue extravasation with righ hemisphere of htMCAO animals was significantly increased as compared with those of sham operated rats (4.52 ± 0.83 versus 1.99 ± 0.42 μg/g, *P *< 0.05). HBO-PC significantly reduced the extravasation of Evans blue as compared with those of htMCAO group (4.52 ± 0.83 versus 2.16 ± 0.47 μg/g, *P *< 0.05; Figure 4).

However HBO-PC failed to improve the mortality rate as compared with htMCAO group (32.5% versus 37.5% in htMCAO group, *P *> 0.05, chi-square test). No animal died in the sham group.

### HBO-PC failed to increase Nrf2 activity and HO-1 expression

**Figure 5:**
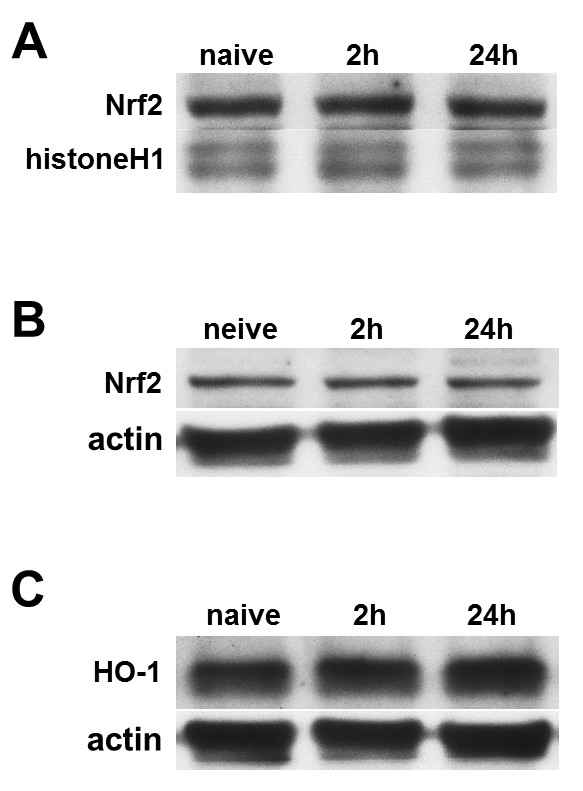
**HBO preconditioning had no effect on Nrf2 and HO-1 regulation before tMCAO**. Representative immunoblots of Nrf2 in nuclear fraction (A), Nrf2 in cytosolic fraction (B), and HO-1 in cytosolic fraction (C) at different time points after the last session of hyperbaric oxygen therapy. There was no statistically significant difference among each time points following HBO-PC.

**Figure 6:**
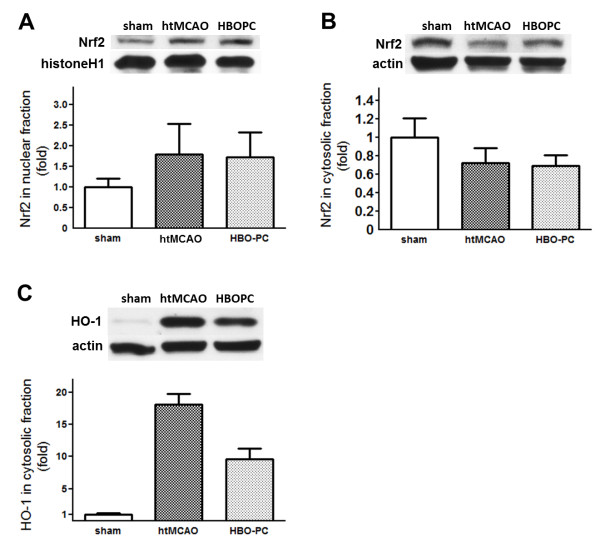
**HBO preconditioning had no effect on Nrf2 regulation, and significantly reduced HO-1 expression**. Representative immunoblots and densitometric analysis of Nrf2 in nuclear fraction (A), Nrf2 in cytosolic fraction (B), and HO-1 in cytosolic fraction (C) at 24 h after MCAO. HBO-PC had no effect on Nrf2 regulation. Furthermore, HO-1 was downregulated by HBO-PC. *P > 0.05 vs. htMCAO, ^#^*P *> 0.05 vs. sham.

Western blot analysis showed that HBO-PC had no effect on Nrf2 regulation. No change was found in the protein expression of Nrf2 in both fractions at 2 or 24 h after the last session of HBO, without subsequent MCAO (Figure 5). Furthermore, there was no significant difference in Nrf2 expression of both fractions at 24 h after MCAO as compared with htMCAO group (Figure 6). Result also shows that no change was found in time-course of HO-1 expression (Figure 5C). However, It is notable that HBO-PC decreased HO-1 at 24 h after MCAO as compared with htMCAO group (Figure 6C).

## Discussion

In this study, we investigated the effect of HBO-PC on HT after transient MCAO and demonstrated that HBO-PC reduced neurological deficit, infarction volume, BBB permeability and HT at 24 h after ischemia. However, no change was found in time-course of Nrf2 in nuclear fraction and HO-1 in cytosolic fraction after repeated HBO exposure, and Nrf2 in nuclear fraction was not upregulated by HBO-PC at 24 h after MCAO. Paradoxically, HBO-PC reduced the protein expression of HO-1 as compared to htMCAO group.

It has been previously suggested that exposure to non-lethal stress can induce protection against subsequent exposure to lethal or more severe stress of a different kind, which is known as cross-tolerance [21]. HBO-PC has been demonstrated to induce cross-tolerance against global cerebral ischemia [22] and focal cerebral ischemia [23]. Under the condition of critical oxidative stress like reperfusion injury, effects of HBO-PC have been attributed not only to reducing ROS/RNS but also to increase of antioxidants which are induced by non-lethal oxidative stress.

Microarray analysis showed that gene expression of Nrf2 and HO-1 was immediately upregulated after HBO administration in human microvascular endothelial cells [14]. In in vivo models, HBO has been shown to induce HO-1 overexpression in the lung [24]. HBO-PC could induce tolerance against ischemia-reperfusion injury by upregulation of HO-1 expression and activity in liver [25] and kidney [26]. Our data did not confirm these findings in the brain of glucose enhanced HT model.

It has been demonstrated that repeated HBO reduced superoxide dismutase and catalase while glutathione peroxidase was up-regulated as compared to single exposure of HBO in blood plasma of patients [27] and the liver of rats [28]. Hence, there is possibility that certain antioxidants may be reduced by repeated exposure of HBO as compared with single exposure. Nrf2 is considered to be activated by ROS/RNS as a non-lethal stress caused by HBO exposure. As a result, repeated preconditioning with HBO down-regulates Nrf2 activation and its downstream effector HO-1 after ischmic insult. On the other hand, several studies measured antioxidants as potential indirect markers of oxidative stress taking place in the brain with stroke [29]. Hemorrhagic transformation and infarction volume of HBO preconditioned group was significantly reduced in this current study, which indicates HO-1 was up-regulated in proportion to the degree of hemorrhagic transformation and infarction. In any case, activated Nrf2/ARE pathway and subsequent HO-1 up-regulation could not be regarded as a cause of reducing HT after repeated HBO exposure in focal ischemia rats.

In this study, hemoglobin leakage in the brain of HBO-PC group was approximately 32% less than in htMCAO group, whereas infarction volume was approximately 52% less. These results indicate that attenuation of HT after HBO-PC can be accountable for reduction in infarction volume. However, Evans blue extravasation in HBO-PC was significantly reduced and approximating to those of the sham group at 24 h after MCAO. This result suggested that HBO-PC is expected to reduce BBB permeability more effectively if observation is undertaken over an extended period. Previous reports demonstrated that HBO-PC suppressed matrix metalloproteinase (MMP) 9 activity after global ischemia [30] and focal ischemia in brain [31]. In the context of HT after cerebral ischemia, MMPs may degrade extracellular matrix and vascular basal lamina, weaken vessels, and predispose them to leakage and rupture. These reports indicate that HBO-PC can reduce HT not only due to the reduction of infarction volume but amelioration of BBB disruption.

HBO-PC did not reduce the mortality rate comapred to htMCAO group despite improvement in neurobehavior tests and HT. It is possible that HBO-PC did not improve systemic complications due to hyperglycemia and MCAO that contributed to the high mortality rates. Systemic complications such as extracellular acidosis have been reported to occur after transient MCAO in hyperglycemic rats [32]. Further study will be needed to investigate the complications which are not directly related to the brain damage but may cause high mortality rates.

HBO-PC has been suggested to reduce oxidative stress, inflammation, and apoptosis in ischemia-reperfusion injury. The result of this study indicates that there are some other mechanisms which contribute to attenuate HT after glucose enhanced ischemia-reperfusion injury in brain. To our knowledge, this is the first investigation that HBO-PC can reduce HT after ischemia. Further studies will be needed to explore the mechanism and to define the optimal regimen of HBO-PC.

## Competing interests

The authors declare that they have no competing interests.

## Authors' contributions

YS conducted most of the animal surgery and wrote the first draft of this paper. RPO provided guidance for surgery and technical support for morphological studies. AM conducted animal behavioral testing. MF assisted in molecular biological studies. JT provided guidance in molecular biological studies. JHZ provided overall supervision. All authors read and approved the final manuscript.
